# Thymoma-associated anti-AMPAR encephalitis with myasthenia gravis: a case report

**DOI:** 10.3389/fimmu.2026.1627951

**Published:** 2026-04-15

**Authors:** Wenbin Teng, Fuzan Wei, Guiqiang Zhang, Shaodan Zhou, Ruiting Hu

**Affiliations:** Department of Neurology, The Minzu Hospital of Guangxi Zhuang Autonomous Region, Nanning, China

**Keywords:** anti-AMPAR antibody, autoimmune encephalitis, myasthenia gravis, paraneoplastic neurological syndrome, thymoma

## Abstract

**Background:**

Thymoma can trigger a spectrum of paraneoplastic neurological disorders, including autoimmune encephalitis (AE) and myasthenia gravis (MG). However, the sequential development of anti-α-amino-3-hydroxy-5-methyl-4-isoxazole-propionic acid receptor (AMPAR) autoimmune encephalitis followed by MG in the same patient is rarely reported.

**Objective:**

To describe the clinical presentation, diagnostic work-up, treatment response, and outcome of a patient who developed thymoma-associated anti-AMPAR encephalitis and later presented with MG.

**Methods:**

We retrospectively reviewed the medical records, imaging findings, laboratory results, and treatment course of a 49-year-old man who was evaluated at our institution. Serum and cerebrospinal fluid (CSF) autoantibodies were screened using cell-based assays and indirect immunofluorescence.

**Results:**

The patient initially presented with subacute cognitive decline, confusion, and behavioral changes. CSF analysis revealed mild lymphocytic pleocytosis. Anti-AMPAR antibodies were positive in both serum and CSF, and contrast-enhanced chest computed tomography (CT) scan showed an anterior mediastinal mass consistent with thymoma. High-dose intravenous methylprednisolone followed by tapering oral prednisone, plasma exchange (PE), and intravenous immunoglobulin (IVIG) and rituximab led to marked neurological improvement. Two months after discharge, he developed left-sided ptosis, diplopia, and dyspnea. Repetitive nerve stimulation showed a decremental response, and serology was positive for acetylcholine receptor (AChR), titin, and ryanodine receptor (RyR) antibodies, establishing the diagnosis of MG. Symptoms improved substantially with prednisone and tacrolimus, and subsequent thymectomy combined with efgartigimod further achieved sustained clinical remission.

**Conclusions:**

This case illustrates that patients with anti-AMPAR encephalitis should be closely monitored for the subsequent emergence of MG, particularly when an underlying thymoma is present. Comprehensive antibody profiling is essential for early recognition and targeted immunotherapy. Long-term immunosuppression combined with tumor-directed treatment may be required to prevent relapses of both disorders and achieve optimal clinical outcomes.

## 1 Introduction

Autoimmune encephalitis (AE) is an immune-mediated inflammatory disorder of the central nervous system (CNS) directed against neuronal antigens. The clinical phenotype typically encompasses subacute memory impairment, psychiatric symptoms, behavioral changes, seizures, altered consciousness, autonomic instability, and movement disorders (1). AE is classified by the target antigen: antibodies directed against intracellular antigens (e.g., Hu, Yo, Ri, Ma2, CV2, amphiphysin, anti-neuronal nuclear antibody type 3 (ANNA-3), Tr, Purkinje cell cytoplasmic antibody type 2 (PCA-2), and glutamic acid decarboxylase (GAD)) and those against cell-surface or synaptic antigens (e.g., N-methyl-D-aspartate receptor (NMDAR), leucine-rich glioma-inactivated 1 (LGI1), gamma-aminobutyric acid-B receptor (GABABR), anti-α-amino-3-hydroxy-5-methyl-4-isoxazole-propionic acid receptor (AMPAR), contactin-associated protein 2 (CASPR2), dipeptidyl-peptidase-like protein 6 (DPPX), and IgLON family member 5 (IgLON5)) (2, 3). This classification is consistent with the Graus criteria (2), which serve as the gold standard for AE diagnosis by linking clinical manifestations to antigen-specific autoantibody profiles. Intracellular antibodies are usually paraneoplastic and associated with cytotoxic T-cell responses, whereas antibodies against surface antigens are more often non-paraneoplastic and mediated by B-cell–dependent mechanisms (4–6). When AE is linked to an underlying tumor, it is termed paraneoplastic AE.

Anti-AMPAR autoimmune encephalitis is a rare autoimmune disorder that targets the AMPA-type glutamate receptor. Two subtypes are recognized based on the specificity of autoantibodies: anti-AMPAR1 (glutamate receptor (GluR1)) and anti-AMPAR2 (GluR2) encephalitis. Anti-AMPAR encephalitis was first characterized by Lai et al. in 2009, with the majority of pathogenic antibodies directed against the GluR2 subunit of the AMPAR (7, 8). AMPARs are heterotetrameric ion channels composed of combinations of GluR1–GluR4 subunits that mediate fast excitatory synaptic transmission and are essential for synaptic plasticity, learning, and memory (9). In the adult CNS, the majority of AMPARs contain the GluR2 subunit, which limits Ca²^+^ permeability and thereby modulates synaptic strength (10). GluR2-containing AMPARs are widely expressed in the limbic system, cerebral cortex, striatum, cerebellum, brainstem, and spinal cord, which aligns with the broad clinical spectrum observed in anti-AMPAR encephalitis (7).

Myasthenia gravis (MG) is an antibody-mediated disorder of neuromuscular transmission. Pathogenic autoantibodies most commonly target the acetylcholine receptor (AChR), but other antigenic targets include muscle-specific tyrosine kinase (MuSK), low-density lipoprotein receptor-related protein 4 (LRP4), and ryanodine receptor (RyR). The coexistence of anti-AMPAR encephalitis and MG is exceedingly rare and suggests a shared autoimmune diathesis. which is often associated with thymic pathology. Here, we describe a patient who presented with anti-AMPAR encephalitis. He was found to have a thymoma and subsequently developed MG. We discuss the diagnostic approach, therapeutic strategy, and outcome to provide practical guidance for clinicians encountering similar cases.

## 2 Case presentation

A 49-year-old man was transferred to our hospital on 7 August 2023 with a 20-day history of progressive behavioral changes and psychotic symptoms. His family reported that, beginning on 18 July 2023, he developed incoherent speech, inability to recognize relatives, and transient disorientation, accompanied by dysarthria, hypersalivation, and unsteady gait. There was no fever, loss of consciousness, or convulsions. On 29 July 2023, he had been admitted to the Fifth People’s Hospital of Nanning, where AE was suspected and symptomatic treatment (including normal saline rehydration and anti-psychotic interventions) was started. As no clinical improvement was observed, he was discharged home, where his symptoms intensified, with the development of anorexic and globally withdrawn. The patient previously denied the history of chronic diseases such as diabetes, coronary heart disease, and nephropathy and denied the history of infectious diseases such as hepatitis and tuberculosis.

### 2.1 Physical and neurological examination

The patient was alert but emotionally blunted and non-communicative, precluding detailed cognitive testing. Pupils were 3 mm, equal, and briskly reactive to light. Extrinsic ocular movements were full; no nystagmus or ptosis was present. Facial symmetry was preserved. He withdrew purposefully to pain, but formal strength testing was limited by poor cooperation. Muscle tone appeared normal. Deep-tendon reflexes were brisk symmetrically, and plantar responses were flexor. Meningeal signs were absent. Sensory examination could not be reliably performed.

### 2.2 Laboratory investigations

Hematology showed leukocytosis with neutrophilia. Inflammatory and hepatic markers were elevated: C-reactive protein (CRP) 51.7 mg·L^-^¹ (normal <10), alanine aminotransferase (ALT) 141 U·L^-^¹ (normal 9-50), and aspartate aminotransferase (AST) 110 U·L^-^¹ (normal 15-40). Serology revealed high immunoglobulin G (IgG) titers to cytomegalovirus (1,241 AU·mL^-^¹) and herpes-simplex-virus type 1 (HSV-1) (2,060 AU·mL^-^¹). Anti-neutrophil cytoplasmic antibody (ANCA) and tumor-screen profiles showed negative results. Stool studies, coagulation tests, and lipid panel had unremarkable findings.

### 2.3 Cerebrospinal fluid

Opening pressure was normal. Cerebrospinal fluid (CSF) contained 6 leukocytes·µL^-^¹ (lymphocyte-predominant), protein 1.08 g·L^-^¹ (normal 0.15-0.45), and normal glucose. Cell-based assays (Credo Diagnostics, Guangzhou) detected anti-AMPAR antibodies in CSF (titer 1:10) and serum (titer 1:30). These titers hold clinical significance. Cell-based assays are the gold standard for detecting pathogenic anti-AMPAR antibodies with high specificity. The concurrent positivity in both CSF and serum confirms the diagnosis of autoimmune encephalitis involving synaptic antigens, effectively ruling out false positivity (2, 11). Screening for anti-NMDAR, LGI1, GABABR, CASPR2, DPPX, and IgLON5 antibodies showed negative results. Oligoclonal bands were absent.

### 2.4 Neuroimaging

**Figure 1:**
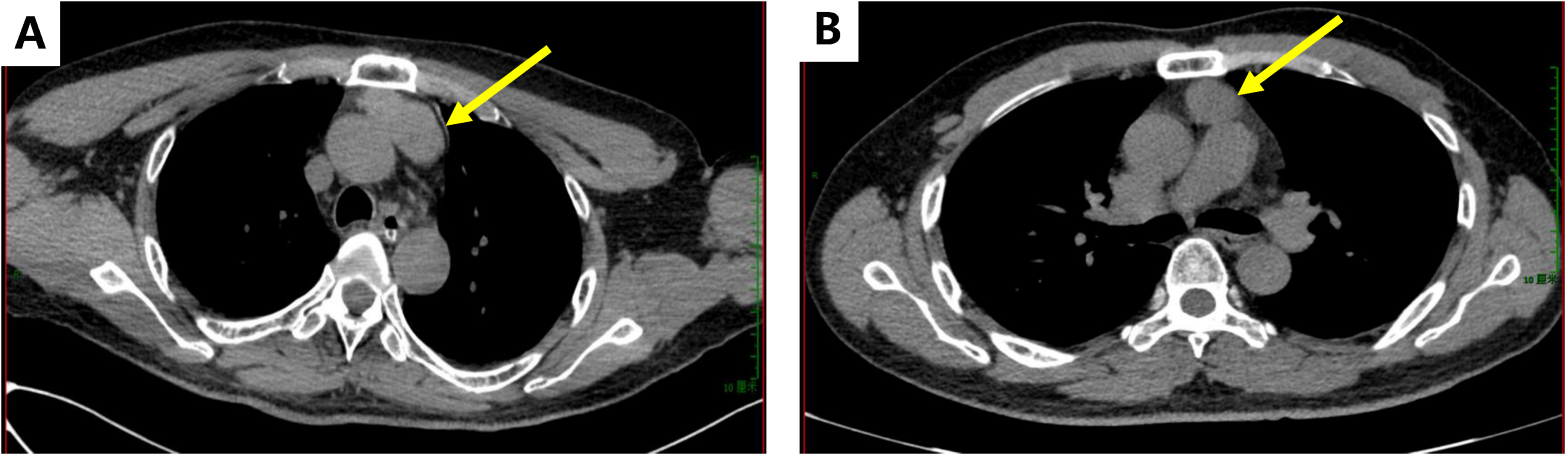
Chest CT. **(A)** Image on August 9, 2023. A patchy soft tissue density shadow appeared in the anterior superior mediastinum, which may be considered as thymoma or thymic hyperplasia. **(B)** Image on November 22, 2023. The mass soft tissue density shadow of the anterior superior mediastinum was thrown to consider the possibility of thymoma, but it was significantly smaller than before.

Brain MRI performed at an outside hospital on 31 July 2023 demonstrated bilateral hippocampal T2/FLAIR hyperintensity with reduced N-acetylaspartate on magnetic resonance spectroscopy, consistent with neuronal injury. Repeat MRI on 10 August 2023 was otherwise normal. Thoracic contrast-enhanced CT on 9 August 2023 revealed a 4.2 × 3.5 cm anterior–superior mediastinal mass compatible with thymoma (**Figure 1A**). Electroencephalography (EEG) showed intermittent generalized theta slowing without epileptiform discharges.

### 2.5 Diagnosis

The final diagnosis was thymoma-associated anti-AMPAR encephalitis.

### 2.6 Treatment and initial outcome

Empirical ganciclovir (0.5 g iv.gtt. every 12 h) and prednisone 20 mg daily were started on admission. From 10 to 18 August, the patient underwent three sessions of plasma exchange (1.0 plasma volume per session). Intravenous immunoglobulin (IVIG) (0.4 g·kg^-^¹·day^-^¹) was given from 23 to 27 August. The patient was initiated on mycophenolate mofetil on 28 August. However, he developed allergic reactions, including pruritus and skin rash. Therefore, tacrolimus (1 mg orally twice daily) was added on 29 August. Rituximab was administered on 30 August (iv 375 mg/m²), but the patient developed abdominal distension and discomfort, so the second dose of rituximab was not given. Broad-spectrum antibiotics and hepatoprotective agents were used as required. By 2 September 2023, the patient’s orientation, speech, and gait had improved substantially; he was discharged home on oral prednisone 60 mg daily (to be tapered) and tacrolimus.

On 13 November 2023, the patient noted the insidious onset of left-sided ptosis without identifiable precipitants. Associated symptoms included a narrowed palpebral fissure, difficulty with voluntary eyelid opening and closure, impaired extraocular movements, and restricted superior and lateral visual fields. The manifestations fluctuated diurnally, being less pronounced in the morning and exacerbated by evening. By 20 November 2023, he had developed generalized fatigue, dyspnea, and dysphagia, necessitating urgent hospitalization.

### 2.7 Admission findings

On admission for MG, the patient was fully alert but disoriented to place and time, with impaired recent memory and calculation. Examination revealed left-sided ptosis and mild limitation of adduction, abduction, and elevation of the left eye. Visual field defects remained confined to the upper and lateral quadrants. Manual muscle testing of the limbs was grade 5/5 proximally and distally. The orbicularis oculi fatigue test was positive. The remainder of the neurological examination showed unremarkable findings.

### 2.8 Laboratory and ancillary investigations

Serum antibodies (Coretti Laboratory) confirmed MG: AChR antibodies 14.439 nmol·L^-^¹ (normal <0.4), titin antibody titer 1:320, RyR antibody titer 1:32. The edrophonium test showed a positive result, and repetitive nerve stimulation testing was precluded by clinical severity. Arterial blood gas analysis demonstrated type II respiratory failure (PaO_2_ 190 mmHg, PaCO_2_ 109 mmHg). Chest CT (22 November 2023) revealed a 5.4 × 4.3 cm anterior mediastinal mass consistent with thymoma (**Figure 1B**). Pulmonary function tests showed severe restrictive ventilatory defect. Due to clinical severity, repetitive nerve stimulation could not be completed.

### 2.9 Myasthenic crisis and ICU management

On 24 November 2023, respiratory failure worsened, prompting transfer to the neurological ICU for endotracheal intubation and mechanical ventilation. The diagnosis of AChR/Titin/RyR antibody-positive MG with myasthenic crisis was established.

Treatment protocol included plasma exchange: five alternate-day sessions; intravenous methylprednisolone: 500 mg daily (26–28 November), tapered to 240 mg daily (29–30 November), and then 120 mg daily (1–3 December), followed by oral prednisone acetate 60 mg daily; pyridostigmine bromide 60 mg every 6 h; empirical broad-spectrum antibiotics and supportive care. The patient was successfully extubated on 12 December 2023 and discharged home on 16 January 2024 with complete resolution of bulbar and limb symptoms.

### 2.10 Relapse and thymectomy

**Figure 2:**
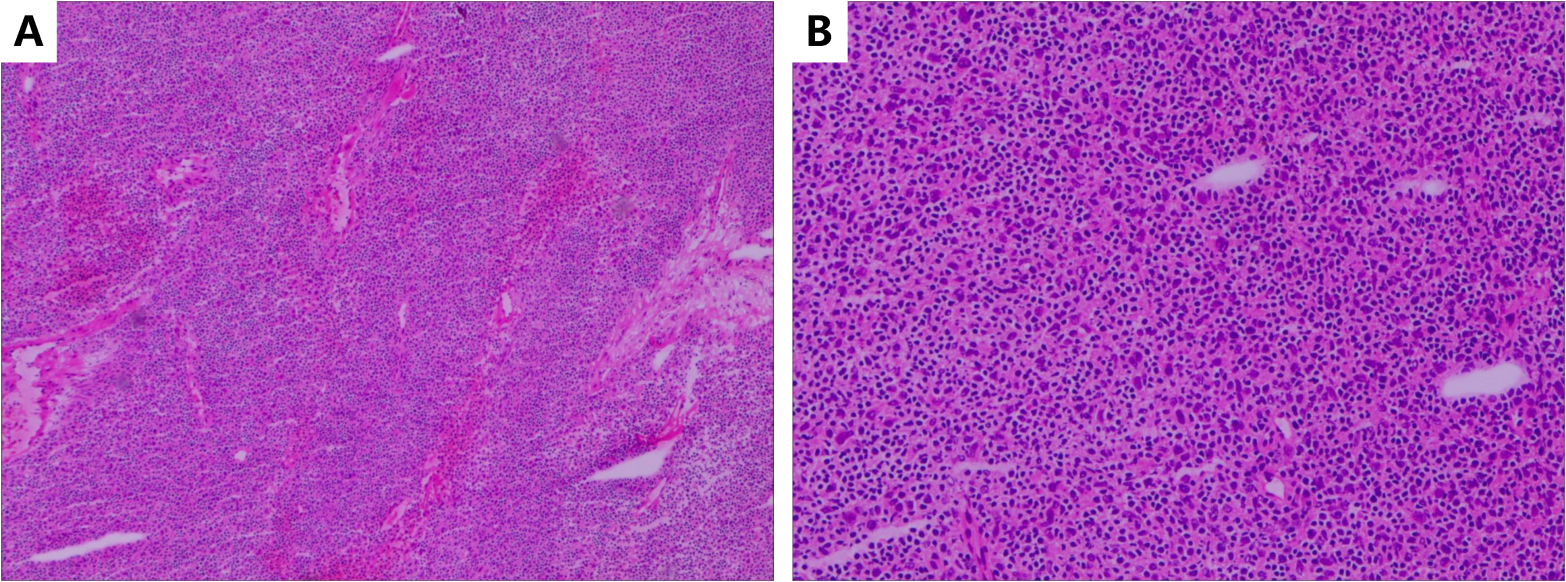
The patient’s postoperative pathology section (hematoxylin–eosin stain: **(A)** ×10, **(B)** × 20).

On 20 September 2024, he was readmitted with 1 week of progressive limb weakness that was refractory to escalated immunosuppressive therapy. On 29 September 2024, he underwent complete thymectomy via left thoracotomy under general anesthesia. A 6 × 5 × 4 cm firm, well-circumscribed mass was removed en bloc. Histopathology confirmed World Health Organization (WHO) type B1 thymoma, Masaoka stage I (pT1N0M0) without capsular invasion. Immunohistochemistry: CK^+^, CK19^+^, CD20^-^, CD3^-^, TdT^-^, CD5^-^, CD117^-^, Ki-67 ≈10% (**Figures 2A, B**).

Postoperative course was complicated by prolonged ventilatory insufficiency; however, after one cycle of efgartigimod (10 mg·kg^-^¹ intravenously), limb strength improved progressively. The patient was weaned from mechanical ventilation on 13 October 2024, achieved an Activities of Daily Living (ADL) score of 1, and was discharged on 25 October 2024 (**Table 1**).

**Table 1:** Key clinical milestones of the patient’s clinical course.

Time point	Clinical event
18 July 2023	Onset of anti-AMPAR encephalitis symptoms (behavioral changes, disorientation)
7 August 2023	Admission to our hospital; initiation of empirical ganciclovir and low-dose prednisone
10–27 August 2023	Initiation of first-line immunotherapy for AE (plasma exchange + IVIG)
28 August 2023	Mycophenolate mofetil (developed pruritus and skin rash)
29 August 2023	Tacrolimus initiated (replaced mycophenolate mofetil due to allergy)
30 August 2023	Rituximab (developed abdominal distension)
2 September 2023	Discharge from hospital with improved AE symptoms
13 November 2023	Onset of MG symptoms (left-sided ptosis, diplopia)
20 November 2023	Urgent hospitalization for MG; formal diagnosis of AChR/Titin/RyR-positive MG
24 November 2023	Development of myasthenic crisis; transfer to Neurological ICU for intubation
26 November–3 December 2023	High-dose glucocorticoid + plasma exchange for myasthenic crisis
16 January 2024	Discharge from hospital with resolved bulbar/limb MG symptoms
20 September 2024	Readmission with MG relapse (progressive limb weakness)
29 September 2024	Complete thymectomy (WHO type B1 thymoma, Masaoka stage I)
October 2024	Efgartigimod administered; progressive improvement in limb strength
25 October 2024	Discharge with sustained clinical remission (ADL score = 1)

AMPAR, anti-α-amino-3-hydroxy-5-methyl-4-isoxazole-propionic acid receptor; AE, autoimmune encephalitis; IVIG, intravenous immunoglobulin; MG, myasthenia gravis; AChR, acetylcholine receptor; RyR, ryanodine receptor; ICU, intensive care unit; WHO, World Health Organization; ADL, activities of daily living.

## 3 Discussion

We report a middle-aged man with thymoma-associated anti-AMPAR encephalitis who developed AChR/Titin/RyR-positive MG 2 months after initial AE treatment and immunotherapy. Repeat brain MRI after MG onset showed no abnormalities. The subsequent development of MG suggests that thymoma-related immune dysregulation may have contributed to the emergence of multiple autoantibodies affecting both central and peripheral synapses. MRI normalization is a common phenomenon in the clinical course of anti-AMPAR encephalitis, especially following early initiation of immunotherapy, and does not require attribution to MG pathophysiology (12, 13). The detection of AChR, titin, and RyR antibodies, together with the presence of a thymoma, supports a diagnosis of thymoma-related PNS involving both AE and MG.

Anti-AMPAR encephalitis and MG are both antibody-mediated disorders of the nervous system, yet their co-occurrence is exceedingly rare (14, 15). The AMPAR is a cell-surface ionotropic glutamate receptor essential for fast excitatory synaptic transmission, synaptic plasticity, learning, and memory (16). In 2009, anti-AMPAR encephalitis was first described in a cohort of 10 patients (17). Subsequent studies demonstrated that exposure of neurons to pathogenic anti-AMPAR antibodies lead to a significant reduction in both the density of AMPAR clusters and the proportion of synaptic sites containing glutamate receptor A 1 (GluA1) and GluA2 subunits, supporting a direct pathogenic role for these antibodies (18). Anti-AMPAR encephalitis is a subtype of AE, which typically presents acutely or subacutely in middle-aged and older adults (12, 19). Characteristic neuroimaging findings include hyperintensities on T2/FLAIR MRI sequences in the medial temporal lobes, particularly the hippocampus and amygdala (2, 20). Clinically, patients commonly present with seizures, cognitive dysfunction, psychiatric disturbances, and speech impairments (11–13). Notably, approximately 70% of cases are paraneoplastic, most frequently associated with lung cancer, thymoma, and breast cancer (11, 12, 17).

MG is characterized by fatigable muscle weakness that improves with rest and is most commonly associated with autoantibodies against the AChR. Importantly, 20%–25% of MG patients have thymoma (21). In this report, we describe a middle-aged man with AMPAR antibody-positive AE and thymoma. His initial presentation included behavioral changes, psychiatric symptoms, memory deficits, and language disturbances. Although immunotherapy led to initial symptom improvement, he developed ocular muscle weakness, generalized fatigue, and respiratory insufficiency 2 months later, culminating in a diagnosis of thymoma-associated anti-AMPAR encephalitis complicated by MG.

Thymomas are known to disrupt central immune tolerance, allowing autoreactive T cells to escape thymic selection and enter the peripheral circulation. This mechanism is thought to contribute to the increased frequency of autoimmune disorders in thymoma patients, including myasthenia gravis and paraneoplastic neurological syndromes (PNS). Up to 40% of thymoma patients develop at least one PNS (22). The presence of multiple neural autoantibodies in our patient confirmed a diagnosis of thymoma-associated PNS. Thymoma-mediated immune dysregulation likely drove the production of diverse autoantibodies targeting both CNS synaptic antigens (AMPAR) and neuromuscular junction antigens (AChR, Titin, and RyR), which provided a unifying mechanism for the sequential development of anti-AMPAR encephalitis and MG in this case.

Although anti-AMPAR encephalitis associated with thymoma has been documented, its co-occurrence with MG remains exceedingly rare. Our patient tested positive for anti-AMPAR antibodies in CSF and serum, as well as for MG-associated antibodies, including AChR-antibody (Ab), Titin-Ab, and RyR-Ab. AChR antibodies are well-established diagnostic markers for MG, detectable in approximately 50%–60% of ocular MG (OMG) and 85%–90% of generalized MG (GMG) cases (23, 24). Titin antibodies are frequently observed in GMG patients with thymoma and are associated with more severe disease, systemic involvement, and respiratory compromise (25, 26). Moreover, Titin antibody titers correlate with disease severity. This correlation suggests that Titin antibody may serve as a biomarker for thymoma. The clinical and serological features of our patient—early-onset GMG, thymoma, and positivity for both AChR-Ab and Titin-Ab—are consistent with previously reported phenotypes.

The etiological management of thymoma-associated AE and MG involves two key strategies (1): treatment of the underlying tumor (e.g., surgical resection, radiotherapy, or chemotherapy) and (2) immunomodulatory therapy (19, 27). For acute or severe presentations, IVIG or PE is recommended, whereas glucocorticoids and immunosuppressants are used for long-term maintenance (19, 27). Rituximab is a second-line especially relevant in antibody-mediated surface antigen AEs like AMPAR, targeting B-cell-driven autoantibody production. On the other hand, efgartigimod is an inhibitor of the neonatal Fc receptor (FcRn). By binding to FcRn, it blocks the recycling of IgG antibodies and promotes their lysosomal degradation, thereby reducing the levels of pathogenic antibodies in the circulation. In the present case, efgartigimod was administered following thymectomy to specifically accelerate the clearance of pathogenic IgG antibodies, including both AChR and AMPAR antibodies. This approach is crucial for the management of refractory myasthenia gravis and for mitigating immune-mediated tissue injury (28, 29). Patients with concurrent AE and MG often experience more severe disease courses and higher relapse rates. Nevertheless, early and aggressive immunotherapy is generally associated with favorable outcomes (21, 30). A minority of patients show limited response to immunotherapy (2). Notably, thymectomy does not completely prevent the onset or recurrence of AE and MG. Luo et al. (15) reported that a patient with MG had AE symptoms in 2 years after thymectomy. Meanwhile, Ouyang et al. (28) reported that a patient with AE and type A thymoma developed MG symptoms 4 months after thymectomy. Neither case showed thymoma recurrence, potentially due to persistent autoreactive T lymphocytes in peripheral blood and the nervous system (14, 29).

Following the diagnosis of AE, tacrolimus was introduced to optimize immunosuppressive therapy; however, the patient subsequently developed MG. One possible explanation may be the absence of long-term maintenance immunotherapy with a stable glucocorticoid regimen after AE discharge. Another possible contributing factor is that the thymoma had not yet been surgically removed at that time, leaving the primary source of immune dysregulation unaddressed. Clinical symptoms were effectively controlled following thymoma resection, multimodal immunotherapy, and biologic-targeted treatment. This outcome supports the potential benefit of long-term immunosuppression in preventing relapse. Importantly, because immunotherapy is effective for both AE and MG, treatment of one condition may concurrently ameliorate the other. From a clinical standpoint, we emphasize the importance of a thorough medical history, along with early lumbar puncture, EEG, MRI, and comprehensive autoimmune antibody screening, to facilitate timely and accurate diagnosis. We further highlight the need for close long-term monitoring of thymoma patients with AE for the subsequent development of MG, and vice versa.

## 4 Conclusion

In summary, thymoma-associated anti-AMPAR encephalitis with concomitant MG is a rare but clinically significant condition. Patients typically exhibit overlapping neurological and neuromuscular symptoms and test positive for multiple autoantibodies targeting both central and peripheral synaptic antigens. While the precise interplay among these immune responses remains to be fully elucidated, thymoma likely serves as a key immunological trigger, promoting broad immune dysregulation and the production of diverse pathogenic autoantibodies. The co-occurrence of these disorders underscores the need for comprehensive autoimmune evaluation in all thymoma patients, including simultaneous screening for both AE and MG-associated autoantibodies. Early and aggressive immunotherapy, combined with timely tumor-directed treatment, is essential for optimal symptom control and improved prognosis.

This rare case provides valuable clinical insights for the diagnosis and management of thymoma-associated anti-AMPAR encephalitis and MG. It highlights the importance of standardized terminology, close long-term monitoring, and a multimodal therapeutic approach combining immunomodulation and tumor resection to achieve sustained clinical remission.

To sum up, this case is relatively rare and lacks guidelines and treatment experience. Firstly, the treatment of this patient was based on the treatment guidelines of anti-AMPAR encephalitis. Then, the treatment was guided by the treatment methods of MG. This finding may offer valuable insights for informing clinical decision-making in the management of such disorders.

## Data Availability

The datasets presented in this article are not readily available because of ethical and privacy restrictions. Requests to access the datasets should be directed to the corresponding author/s.
